# Does rotavirus turn on type 1 diabetes?

**DOI:** 10.1371/journal.ppat.1007965

**Published:** 2019-10-10

**Authors:** Leonard C. Harrison, Kirsten P. Perrett, Kim Jachno, Terry M. Nolan, Margo C. Honeyman

**Affiliations:** 1 Walter and Eliza Hall Institute for Medical Research, University of Melbourne, Melbourne, Victoria, Australia; 2 Vaccine and Immunization Research Group, Murdoch Children’s Research Institute and the Melbourne School of Population and Global Health, University of Melbourne, Parkville, Victoria, Australia; 3 Clinical Epidemiology and Biostatistics Unit, Murdoch Children's Research Institute, Parkville, Victoria, Australia; University of Wisconsin Madison, UNITED STATES

## Introduction

Rotavirus (RV) remains the major cause of infantile gastroenteritis worldwide, although the advent of vaccination has substantially decreased associated mortality [1]. Recently, we observed a 15% decrease in the incidence of type 1 diabetes (T1D) in Australian 0–4-year-old children following the introduction of RV vaccination [2, 3], suggesting that RV vaccination could contribute to the primary prevention of this autoimmune disease. This finding builds on our human and animal studies implicating RV in the development of T1D in genetically susceptible children.

## The first clue—Molecular mimicry

**Fig 1:**
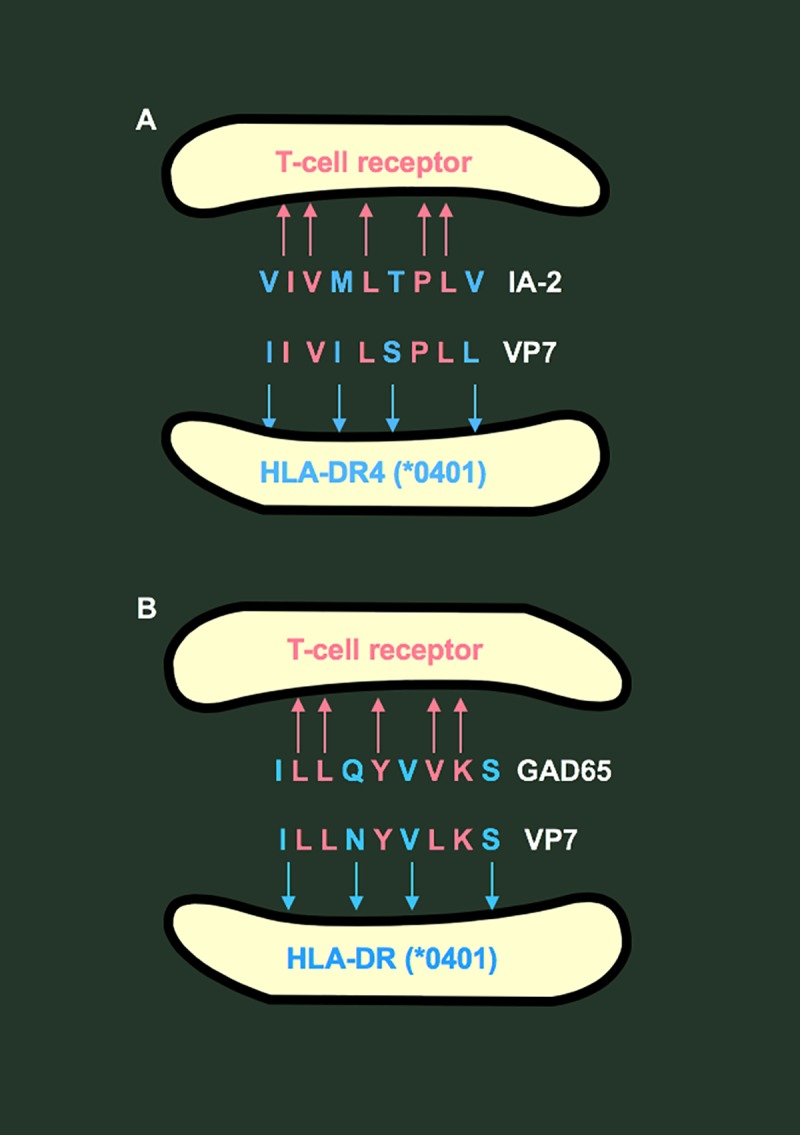
Mimicry between amino acid sequences in islet autoantigens IA-2 (A) and GAD65 (B) and rotavirus genotype 3 viral protein 7 (VP7).

T1D is a polygenic autoimmune disease shaped by environmental modifiers that results in destruction of insulin-secreting beta cells in the pancreatic islets. Approximately 50% of the familial risk of T1D is attributed to the human leukocyte antigen (HLA) gene region on Chromosome 6p21. In 1994, during a short sabbatical in the laboratory of Dr. Luciano Adorini in Milan, we (LCH and MCH) purified HLA class II proteins that confer high risk for T1D and the equivalent single major histocompatibility complex (MHC) protein, I-Ag7, of the nonobese diabetic (NOD) mouse, a model of T1D. HLA class II proteins bind and present peptides (epitopes) to T cell receptors on CD4^+^ T cells. We measured binding to purified HLA/MHC proteins of multiple overlapping 10- to 15-mer synthetic peptides, including from the islet autoantigens proinsulin, IA-2 and GAD65, to define peptide binding “motifs” and identify candidate epitopes for “diabetogenic” T cells [4–9]. In testing the ability of peptides to stimulate blood T cells from islet autoantibody-positive T1D relatives, we identified a dominant epitope, VIVMLTPLVEDGVKQC (amino acid [aa] 805–820) in IA-2, which had 56% identity and 100% similarity over 9 aa with a sequence (aa 40–48) in the major immunogenic viral protein 7 (VP7) outer-capsid protein of human RV serotype genotype 3 (G3), strain P (Fig 1A) [6,7]. Both peptides bound to human leukocyte antigen-D-related (HLA-DR4) (*0401), which confers risk for T1D, and were recognized by the same T cell receptor [9], consistent with functional molecular mimicry.

In addition to mimicry with IA-2, a neighbouring sequence in VP7 (aa 17–25) (Fig 1B) had 78% identity and 100% similarity over 9 aa with a known HLA-DR4-restricted T-cell epitope in GAD65 [7]. Moreover, these IA-2 and GAD65 DR4-restricted epitopes encompassed T-cell epitopes for HLA class I–restricted CD8^+^ T cells in T1D [10], for which we coined the term “combitope.” We hypothesized that T cells activated by RV could trigger or exacerbate islet autoimmunity by molecular mimicry with IA-2 or GAD65 epitopes [7–9]. Although molecular mimicry is intriguing, its role as a causal mechanism in human disease can only be inferred. Australian surveillance data [11] show that the prevalence of RV G3 strains increased slightly along with an increase in strain diversity in the post-RV vaccine era, but G3 remains a minor component of disease-causing RV strains. These data do not indicate any correspondence between rates of G3 infection and T1D.

## Serum islet autoantibodies are associated with RV infection

To gain more direct evidence for a role of RV in T1D, we sought a temporal association between islet autoantibodies and RV infection in 360 children at genetic risk for T1D who were monitored serially from birth [12]. In 24 children in whom islet autoantibodies were first detected or increased in concentration, RV IgG or IgA antibodies were temporally associated with autoantibodies to IA-2, insulin, and GAD65 in 86%, 62%, and 50% of cases, respectively, confirmed by random permutation analysis to be highly significant. It would be important to determine whether islet autoantibodies cross-react with RV, but to our knowledge, this has not yet been investigated.

## RV infection induces pancreatic pathology

**Fig 2:**
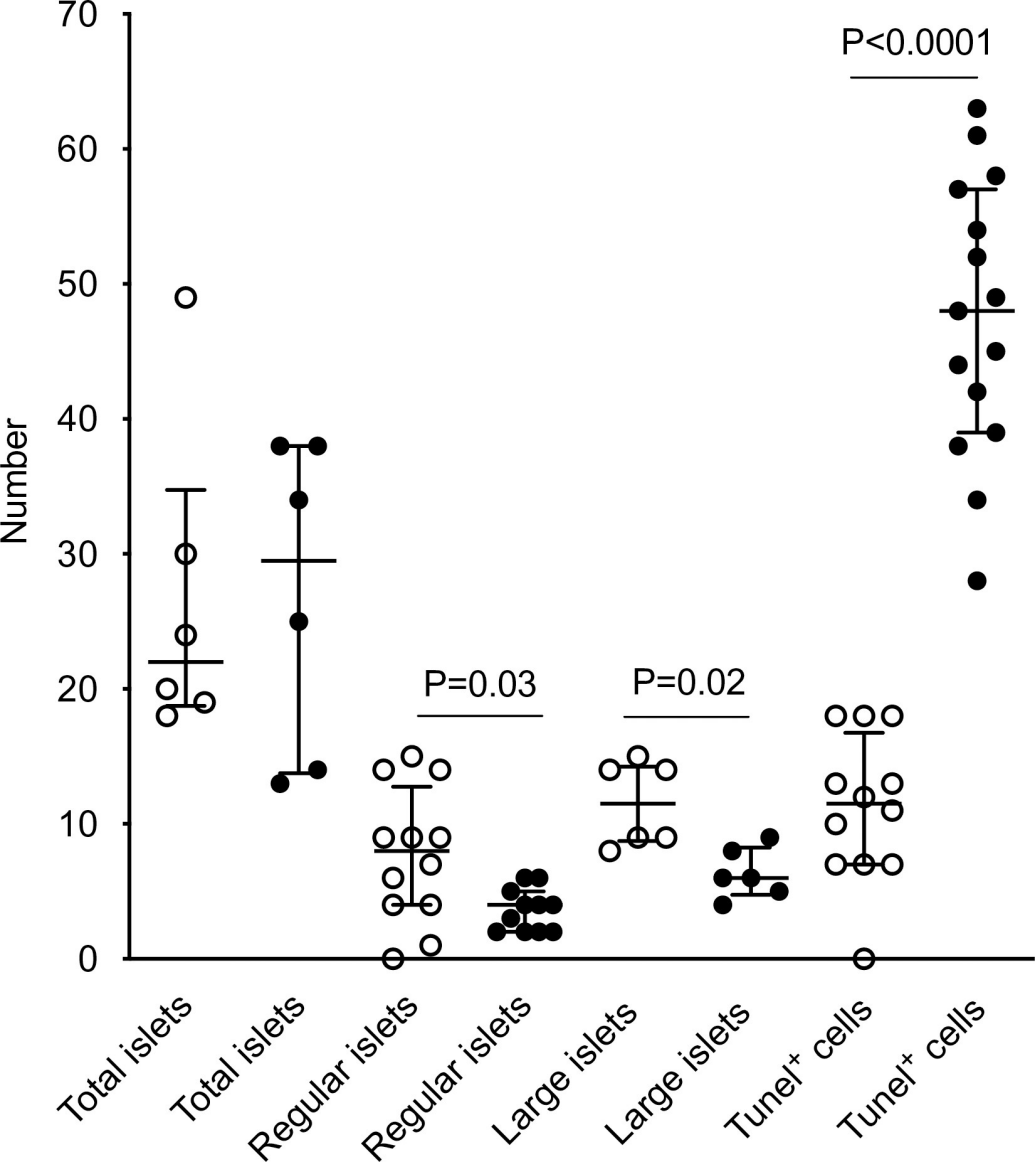
Decrease in islet regularity and size associated with global pancreatic apoptosis (Tunel^+^) 2–3 days after oral inoculation of weaned C57Bl/6 mice with rhesus RV (filled circles) or mock control (open circles). Data are median with interquartile range. Groups were compared by unpaired Mann–Whitney test. Adapted from Ref. [16]. RV, rotavirus.

Evidence that RV induces pancreas pathology is likely to be most relevant to a role for RV in T1D. We showed that rhesus RV infected the islets of NOD mice and other species and that human RV infected monkey islets [13]. This was not surprising because reoviruses (which, like RVs, are members of the Reoviridae family of double-stranded [ds] RNA viruses) had been shown to infect mouse pancreatic beta cells, resulting in diabetes [14], and up-regulate MHC class I protein expression and induce cytopathic effects in beta cells of human islets [15]. The effects of RV on the pancreas were striking after we orally inoculated C57Bl/6 mice at weaning with rhesus RV [16], which is closely related to human RVs and infects mouse islets in vitro [12]. Two phases of mild, transient hyperglycemia were observed beginning 2 and 8 days after inoculation. In the first, widespread apoptosis of pancreatic cells was associated with decreased islet regularity, size, and insulin production (Fig 2), but virus was not detected in the pancreas. These effects did not occur in mice deficient for Toll-like receptor (TLR)3, which is triggered by dsRNA. By the second phase, pancreas mass and islet size had recovered, associated with widespread cellular proliferation in islets and exocrine pancreas, but many islets remained irregular in size. Viral antigen was then detected in the pancreas for several days, during which time it was positively correlated with mild hyperglycemia. In summary, a single RV infection had a dramatic effect to induce apoptosis in the whole pancreas, initially TLR3 mediated, followed by regeneration with residual damage to the islets. Further investigations may reveal whether RV-induced pancreas injury can trigger islet autoimmunity on a susceptible genetic background, perhaps requiring repeated RV infections as experienced by unvaccinated children. Apart from genetic background, timing of infection may be another important factor. In NOD mice that are genetically prone to immune dysregulation, RV infection in the neonatal period prevented diabetes [17, 18], whereas infection at weaning [18] or in established diabetes [19] had the opposite effect to accelerate diabetes development. A caveat on these studies is that findings in heterologous systems, in which the species origins of RV and pancreas differ, e.g., rhesus RV on mouse pancreas, may not necessarily extrapolate to outcomes in human infants. Overall, however, it is clear that RVs induce pancreas pathology in animal models.

In his seminal description of pancreas pathology, Gepts [20] noted a decrease in the size of the pancreas in T1D. Nearly 50 years passed before this key observation was confirmed in relatives at risk for T1D [21]. Whether decreased pancreatic size has a genetic or acquired basis is unclear, but it implicates the total pancreas and not just the islets in the pathology of T1D and is reminiscent of our observations in C57/BL6 mice infected with RV [16].

## Environmental factors promote the rise in incidence of T1D

The incidence of childhood T1D around the world began to increase in the second half of the last century [22]. In Australia, we found that this was accounted for by an increase in cases carrying lower-risk HLA genes (DR 3,3; 4,4; 3,X; 4,X), whereas the absolute number of highest-risk DR3,4 cases did not change [23]. The increased penetrance of lower-risk HLA genes is consistent with a role for environmental factors in promoting disease pathogenesis. An interesting observation in the Australian context that may be relevant to RV as a candidate environmental factor relates to the practice of mothers “rooming-in” with their newborns. This was introduced in the 1970s and entailed mother and baby remaining together rather than separating the baby to a communal nursery at night. RV was prevalent in nurseries, and the change to rooming-in would have altered the timing of exposure to RV, delaying it until later in the first year of life when, based on NOD mouse studies [17–19], RV might promote rather than retard development of diabetes.

## Population-level data suggest RV vaccination may be associated with a decrease in the incidence of T1D

The live oral RV vaccines Rotarix (G1P[8], monovalent, GSK) and RotaTeq (G1-4, P[8], pentavalent, Merck) were introduced into the Australian National Immunisation Program in 2007 and had high uptake (http://www.ncirs.org.au/sites/default/files/2018-11/Rotavirus-history-August-2017.pdf) with a substantial herd immunity effect [24]. We hypothesized that if natural infection with RV was a causative factor in T1D, then RV vaccination would alter the incidence of disease. In the first scenario, the vaccine might elicit T-cell immunity in HLA-DR4 individuals to cross-reactive IA-2 or GAD65 epitopes (Fig 1). In a worst case, this could increase the incidence of T1D. The IA-2 and GAD65 mimicry we identified [7–9] was in VP7 of RV serotype G3, which is only present in pentavalent RotaTeq, but the VP7 sequences of G1, G2, G3, and G4 are more than 90% identical [25], and the sequence in G3 that mimics the GAD65 epitope is similar in the G1 strain of monovalent Rotarix. However, the same can’t be said of the IA-2 sequence, which is mimicked only in G3. We therefore suggest that RotaTeq has the greatest potential to elicit cross-reactive immunity to epitopes in both islet autoantigens. In the second scenario, if RV was implicated in T1D by a direct effect on the pancreas, which seems more plausible, then vaccination could prove protective in vaccine recipients or indirectly through herd immunity.

As a prelude to an individual-level case-control study, we used publicly available data to perform interrupted time-series analysis of the incidence of T1D in Australian children in the 8 years before and after introduction of oral RV vaccine [2, 3]. In that 16-year period, 16,159 new cases of T1D were recorded among children aged 0 to 14 years, equating to a mean rate of 24.4 (95% CI 22.4–26.7) cases per 100,000 children. In children aged 0 to 4 years, the number of incident cases decreased by 15% (rate ratio, 0.85 [95% CI 0.75–0.97]; *P* = 0.02) after the RV vaccine was introduced. The pre- and postintervention patterns did not change over time, and no differences were observed in children aged 5–9 and 10–14 years. This was the first report of a decrease in the incidence of T1D in children born after introduction of RV vaccine.

Our finding was recently confirmed in a large historical cohort study by Rogers and colleagues [26], who published an analysis of T1D incidence in relation to RV vaccination using private insurance data 2001 to 2017 from nearly 1.5 million US children. In children born in 2006 to 2017, more than 540,000 received all three doses of RV vaccine, nearly 141,000 received at least one dose, and 246,600 were not vaccinated. The incidence of T1D in fully vaccinated children was 12.2 cases per 100,000 person years, compared to 20.6 in those unvaccinated, i.e., a 41% difference. Importantly, the incidence was not altered in children who were only partially vaccinated. In fully vaccinated children, pentavalent RotaTeq vaccine was used in 83% of cases. The reduction in the risk of T1D was 33% among vaccine recipients as a group (95% CI 17%–46%) and, interestingly, was greater for pentavalent RotaTeq vaccine (37%) than for monovalent Rotarix vaccine (27%) when regressed simultaneously in the survival analysis. This difference may hold a clue to the mechanism of protection. We extrapolate from Roczo-Farkas and colleagues [11] that the pentavalent vaccine was less commonly used in Australian cases, although, to our knowledge, there is no evidence that either vaccine has superior effectiveness against RV infection.

A Finnish population-based cohort study of 495 T1D cases born in 2009 and followed for 5 years was inconclusive regarding an association between RV vaccination and T1D or celiac disease risk [27], but it examined a relatively small number of cases over a short time frame. In addition, the effect of RV vaccination could vary by geographical location due to genetic and environmental differences. Environmental factors promoting the increase in T1D incidence on particular genetic backgrounds [23] are likely to be ubiquitous and multiple. That RV may be one such factor is supported by several lines of evidence described earlier, to which we can now add an association between RV vaccination and a decrease in T1D incidence. This may be the first clear example of primary prevention of T1D. If ecologic and/or cohort/case-control studies in other jurisdictions are confirmatory, it will be important to identify, if possible, which children are most likely to be protected by RV vaccination. Epidemiological studies alone cannot establish a causal relationship between RV and T1D but are an impetus to understand disease mechanisms and directly demonstrate whether RV infects the human pancreas prior to the onset of islet autoimmunity or T1D, a challenge that may now be feasible through organ donor initiatives such as nPOD (www.jdrfnpod.org).
